# Tailored Sample Mounting for Light-Sheet Fluorescence Microscopy of Clarified Specimens by Polydimethylsiloxane Casting

**DOI:** 10.3389/fnana.2019.00035

**Published:** 2019-03-27

**Authors:** Antonino Paolo Di Giovanna, Caterina Credi, Alessandra Franceschini, Marie Caroline Müllenbroich, Ludovico Silvestri, Francesco Saverio Pavone

**Affiliations:** ^1^European Laboratory for Non-Linear Spectroscopy, University of Florence, Sesto Fiorentino, Italy; ^2^Department of Information Engineering (DINFO), University of Florence, Florence, Italy; ^3^School of Physics and Astronomy, University of Glasgow, Glasgow, Scotland; ^4^National Institute of Optics, National Research Council, Florence, Italy; ^5^Department of Physics and Astronomy, University of Florence, Sesto Fiorentino, Italy

**Keywords:** light-sheet microscopy, tissue clearing, brain, sample mounting, PDMS

## Abstract

The combination of biological tissue clearing methods with light-sheet fluorescence microscopy (LSFM) allows acquiring images of specific biological structures of interest at whole organ scale and microscopic resolution. Differently to classical epifluorescence techniques, where the sample is cut into slices, LSFM preserves the whole organ architecture, which is of particular relevance for investigations of long-range neuronal circuits. This imaging modality comes with the need of new protocols for sample mounting. Gel matrix, hooks, tips, glues, and quartz cuvettes have been used to keep whole rodent organs in place during image acquisitions. The last one has the advantage of avoiding sample damage and optical aberrations when using a quartz refractive index (RI) matching solution. However, commercially available quartz cuvettes for such large samples are expensive. We propose the use of polydimethylsiloxane (PDMS) for creating tailor-made cuvettes for sample holding. For validation, we compared PDMS and quartz cuvettes by measuring light transmittance and performing whole mouse-brain imaging with LSFM. Moreover, imaging can be performed using an inexpensive RI matching solution, which further reduces the cost of the imaging process. Worth of note, the RI matching solution used in combination with PDMS leads to a moderate expansion of the sample with respect to its original size, which may represent an advantage when investigating small components, such as neuronal processes. Overall, we found the use of custom-made PDMS cuvettes advantageous in term of cost, image quality, or preservation of sample integrity with respect to other whole-mouse brain mounting strategies adopted for LSFM.

## Introduction

Light-sheet fluorescence microscopy (LSFM) has allowed high-throughput imaging of large intact biological samples at subcellular resolution (Voie et al., 1993; Engelbrecht and Stelzer, 2006; Dodt et al., 2007). Conversely to other fluorescence microscopy techniques, in LSFM the sample is not cut into slices but instead, optical planes are acquired from the intact cleared sample. This modality of image acquisition has forced experimentalists to create new strategies for sample mounting. Noted by Reynaud et al. (2015): “Light-sheet microscopy offers the possibility of designing microscopes for specific, often three-dimensional (3D), samples, but this can mean doing away with the coverslip and, with it, many well-established protocols for sample mounting.” Ranging from custom-made to commercially available light-sheet microscopes, different mounting techniques for imaging of large samples have been designed (Silvestri et al., 2013; Reynaud et al., 2015). One of these consists in embedding the sample in a matrix gel, such as agarose. The use of agarose gel has been successful in immobilizing embryos and zebrafish larva for *in vivo* imaging (Udan et al., 2014; Müllenbroich et al., 2018). However, for larger specimens, such as mouse brain, the agarose concentration needed for creating a stable matrix has a detrimental impact on the light sheet quality, because of optical aberrations caused by the refractive index (RI) inhomogeneity between the agarose sample holder and the mounting medium. To avoid any RI mismatch caused by the presence of the holder along the light path, the specimen can be fastened with a hook or plunged on a tipped mounting plate. Both methods, however, inevitably damage the samples. Alternatively, specimens can be glued on a disk made of agarose or on a coverslip. In the latter cases, the presence of the glue hinders a proper propagation of the light sheet in the glued volume, resulting in poor image quality. A customized quartz cuvette has been used to hold mouse brain incubated in 2,2′thiodiethanol (TDE)/phosphate buffered saline (PBS) solution with a RI close to the quartz (Costantini et al., 2015). Such an option solves both the problem of sample damaging and RI inhomogeneity. On the other hand, since in this case the sample is held in place by contact with cuvette walls, tailor-made cuvettes fitting samples size are needed. Because of the elevated cost of customized quartz cuvettes, we looked for alternative low-cost compounds to be used. In order to make a customizable holder, the ideal material should be of low viscosity before curing, but rigid enough after the curing step, other than optically transparent. We also paid attention to the handling properties, considering toxicity and simplicity of the curing procedure. Among possible options, UV-curable resins are characterized by low viscosity and high strength after curing. However, the need for UV transparent molds and appropriate UV irradiation must be taken into consideration and may result in increasing technical complexity. Moreover, high volume shrinkage occurs after curing, resulting in a possible build-up of internal stresses, the formation of defects as well as dimensional changes. Because of these constraints, we ruled out UV-curable resins and focused instead on thermo-curable compounds. We found polydimethylsiloxane (PDMS), characterized by optical transparency in the UV-visible region and chemical inertness, as potential material for creating cheap custom-made sample holders. In fact, PDMS has been already used to replicate high aspect ratio microstructured masters (Comina et al., 2014). The PDMS cuvette was realized combining standard micromachining with polymer casting molding processes, which involve casting of material against a mold having the negative shape required for obtaining the final object.

Thanks to its low viscosity, PDMS was easily poured in 1 mm narrow fissures to replicate the thickness of cuvette walls previously used in our LSFM set-up. Furthermore, despite being an elastomeric polymer, PDMS cuvettes resulted in mechanically stable to be mounted firmly in the microscope setup and moved without any fluctuation during sample scanning. Light transmittance curves of CLARITY treated brain slices placed in quartz and PDMS cuvettes revealed comparable properties between the two materials, which encouraged the use of PDMS as a support for whole mouse brain imaging. Whole brain acquisition confirmed an optimal image quality using PDMS cuvettes. Indeed, semi-automatic neurite tracing was possible from the PDMS-mounted sample and was comparable with tracing of the same neuronal processes retrieved from whole brain acquisitions using a quartz cuvette. Due to the lower TDE/PBS ratio used in association with PDMS cuvettes, the sample results expanded with respect to its original size. This feature can be advantageous in term of image resolution. Approaches exploiting sample expansion to achieve resolution beyond the optical limit have in fact been described and are known as expansion microscopy (Chen et al., 2015). Nevertheless, when performing whole mouse brain acquisition with LSFM, particular attention must be paid to the volume of data generated, which exceeds the data usually produced routinely with other microscopy techniques by orders of magnitude (Schmid et al., 2013; Reynaud et al., 2015). We point out, however, that the expansion resulting from our sample treatment is less prominent with respect to that obtained with expansion microscopy approaches described in the literature. This feature is better aligned with requirements for data storage and computation analysis resources available in laboratories, making the suggested methodology a good compromise between the data volume produced and the increase in image resolution.

## Materials and Methods

### Fabrication of PDMS Cuvettes

**Figure 1:**
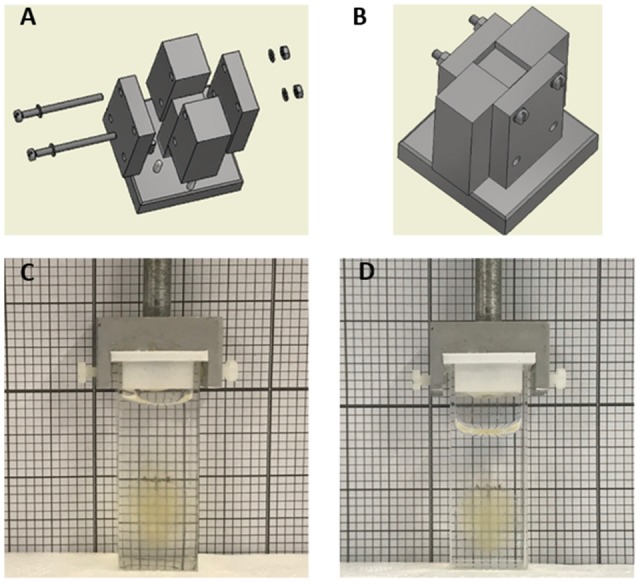
**(A)** Exploded and **(B)** assembled views of the aluminum mold realized by standard machining processes. The modularity design of the mold, consisting of four side parts and a central part assembled on a single platform, was engineered to easily remove the polydimethylsiloxane (PDMS) part at the end of the curing process. **(C,D)** Whole cleared mouse brain in PDMS **(C)** and quartz **(D)** cuvette in 40% and 68% TDE/phosphate buffered saline (PBS), respectively.

Aluminum (Al) casting frames were realized by implementing a standard milling process. Post-processing steps involving surface finishing were implemented to smooth the internal surfaces of the mold and to give to aluminum facets a mirror-like surface finish. To this end, surface roughness was reduced from about 12.5 μm to 0.8 μm by using 600-grit sandpaper. As shown in Figures 1A,B, the final mold design was composed of tightly assembling four side parts and a central part on a platform. Such design enables to easily remove the cuvette at the end of the fabrication process. The overall dimensions of the assembled mold were 27 × 22 × 65 mm, placed on a base of 40 × 40 × 10 mm. In order to obtain cuvettes with thin walls, the distance between the central and the side surfaces was 1 mm (Supplementary Data Sheet 1).

PDMS (Sylgard 184, Dow Corning) was mixed in a 10:1 mass ratio of prepolymer to curing agent. The resulting mixture was centrifuged for 5 min, vacuum degassed to remove air bubbles and poured in the Al mold. One-hour vacuum degassing was repeated to ensure that the solution totally filled the stamp. Crosslinking of the PDMS was achieved after 12 h at room temperature and 2 h at 70°C. Once returned to room temperature, the PDMS cuvette was released by disassembling the different parts composing the mold and left again at 70°C for 2 h to ensure that the PDMS was properly cured at the PDMS/air interface. Finally, to prevent uncured PDMS from sticking to the surfaces and facilitate PDMS cuvette extraction, the central piece of the Al mold was functionalized with perfluorodecyltriethoxysilane (PFDTES) through vapor phase silanisation.

### Mouse Perfusions and Whole Brain Clearing With CLARITY-TDE

After deep anesthesia with isoflurane inhalation, wild type C57 and *Thy-1*-GFP-M (Feng et al., 2000) mice were transcardially perfused first with 20–30 ml of 0.01 M (PBS) solution (pH 7.6) and then with 60 ml of 4% (w/v) paraformaldehyde (PFA) in PBS. The brains were extracted and incubated overnight in 4% PFA in PBS at 4°C. The following day, the brains were rinsed three times in PBS.

The fixed *Thy-1*-GFP-M mouse brain was incubated in Hydrogel solution [4% (wt/vol) acrylamide, 0.05% (wt/vol) bisacrylamide, 0.25% (wt/vol) VA044] in 0.01 M PBS at 4°C for 3 days. Samples were degassed and incubated at 37°C for 3 h to allow hydrogel polymerization. Subsequently, brains were extracted from the polymerized gel and incubated in clearing solution [sodium borate buffer 200 mM, 4% (wt/vol) sodium dodecyl sulfate, pH 8.5] at 37°C for 1 month while gently shaking. The samples were then washed with PBST (0.1% TritonX in 1× PBS) twice for 24 h each at room temperature. The samples were then sequentially incubated in 20%, 40%, 68% TDE in PBS (RI 1.37, 1.41, 1.46 respectively), each for 1 day at room temperature with gentle oscillation before imaging.

All experimental protocols involving animals were designed in accordance with the laws of the Italian Ministry of Health. All experimental protocols were approved by the Italian Ministry of Health.

### Measurements of Light Transmittance and Linear Expansion

A PFA-fixed C57 mouse brain was cut into 1 mm coronal slices using a mouse brain slicer (Alto mouse brain coronal matrices, CellPoint Scientific, MD, USA). The 1 mm thick slices were incubated in 10 ml Hydrogel solution [4% (wt/vol) acrylamide, 0.05% (wt/vol) bisacrilamide, 0.25% (wt/vol) VA044] in 0.01 M PBS at 4°C for 1 day. Samples were degassed and incubated at 37°C for 3 h to allow hydrogel polymerization. Subsequently, the slices were carefully extracted from the polymerized gel and incubated in clearing solution [sodium borate buffer 200 mM, 4% (wt/vol) sodium dodecyl sulfate, pH 8.5] at room temperature for 3 days with gently shaking. The samples were then washed with PBST (0.1% TritonX in 1 × PBS) for 24 h at room temperature. The samples were then sequentially incubated in 20%, 40%, 68% TDE in PBS, each for 1 h at room temperature with gentle oscillation. After each step, the transmittance was measured in PDMS and quartz cuvettes (3/Q/15/TW, Starna Scientific Ltd., Ilford, UK) using a spectrophotometer (Jasco V-560, Jasco international co., Japan).

For sample expansion measurements, top view photos of PFA-fixed 1 mm thick mouse brain slices were taken before clearing and after clearing in different percentage of TDE/PBS solutions. From the taken photos the relative areas were measured with ImageJ/Fiji^1^. The linear expansion was quantified normalizing the area of each cleared slice with the corresponding area before clearing and calculation the square root of that quotient.

### Imaging of Biological Samples With LSFM

Whole brains were imaged using a custom-made light-sheet microscope described in Müllenbroich et al. (2015). The light sheet was generated using a laser beam scanned by a galvanometric mirror (6220H, Cambridge Technology, MA, USA); confocality was achieved by synchronizing the galvo scanner with the line read-out of the sCMOS camera (Orca Flash4.0, Hamamatsu Photonics, Japan). The laser light was provided by a diode laser (Excelsior 488, Spectra Physics) and an acousto-optic tunable filter (AOTFnC- 400.650-TN, AA Opto-Electronic, France) was used to regulate laser power. The excitation wavelengths were *λ* = 561 nm for TRITC and *λ* = 491 nm for GFP and fluorescein. The excitation objective was a 10×, 0.3 NA Plan Fluor from Nikon, while the detection objective was a 10×, 0.6 NA Plan Apochromat from Olympus. The latter had a correction collar for the RI of the immersion solution, ranging from 1.33 to 1.52. Excitation objective was largely underfilled, with an effective NA of about 0.02. At such low NAs, the effects of RI mismatch are very small, and thus even an air objective can perform relatively well (Booth et al., 1998). The sample was acquired first using a PDMS cuvette containing 40% TDE/PBS. After 1-day incubation in 68% TDE/PBS, the same sample was acquired with quartz cuvette filled with the same TDE/PBS ratio. Both PDMS and quartz cuvette were placed in a custom-made chamber filled with the mounting medium (40% or 68% TDE/PBS for PDMS and quartz cuvette respectively). RI correction of the detection objective and focus adjustment were carefully carried out before each imaging session. The cuvettes were mounted on a motorized x-, y-, z-, θ-stage (M-122.2DD and M-116.DG, Physik Instrumente, Germany), which allowed free 3-D motion and rotation. Stacks were acquired with a z-step of 2 μm and a xy resolution resulting from the setup configuration of 0.65 μm, with a field of view of 1.3 × 1.3 mm. The microscope was controlled *via* custom-written LabVIEW code (National Instruments), which coordinated the galvo scanners, the rolling shutter, and the stack acquisition.

### Point Spread Function Measurements

For evaluation of optical resolution, we embedded fluorescent beads (FluoSpheres Fluorescent Microspheres, diameter 110 nm, Thermo Fisher Scientific) at a dilution of 1:40,000 in 2% Agar in PBS. After solidification, the agar containing the fluorescent beads was incubated in TDE 40% in PBS or TDE 68% in PBS overnight. The agar, along with the mounting medium, was placed in the PDMS or quartz cuvette. The latter was then positioned in the chamber of the microscope filled with the same medium. Point spread functions and full-width-at-half-maximum (FWHM) in both lateral and axial dimensions were extracted from the microscope images using Huygens software (Scientific Volume Imaging, Netherlands).

### Measurement of Laser Beam Width

To estimate the width of the Gaussian laser beam used for imaging, solutions of TDE 40% and TDE 68% in PBS containing fluorescein were used. These solutions were put in PDMS or Quartz cuvette, with the chamber of the microscope containing the respective medium without fluorescein. Images of the fluorescence resulting from the beam passing through the fluorescent solution were analyzed with a custom-written code (MatLab) to extract a profile of the laser beams. From the obtained profiles, the beam waist (W_0_) and the Rayleigh range (Z_R_) were calculated.

### Image Stitching and 3D Rendering

LSFM acquisition generates a series of 3D stacks which need to be stitched for whole brain rendering. We use the ZetaStitcher software available at https://github.com/lens-biophotonics/ZetaStitcher for image stitching. Three-dimensional renderings of ROIs were produced with the Amira Voltex function of Amira 5.3 software.

### SBR and 3D Measurements of Euclidean Distance Between Neurons and Dendrite Length

In order to evaluate the signal-to-background ratio (SBR) the maximum and the minimum values of the intensity profiles of lines passing through neuronal somas of dendrites were taken respectively as values of signal and background. The same neurons and neuronal processes were used for comparison of the images obtained with PDMS and quartz cuvette.

Euclidean distance between neurons and dendrite length were measured from LSFM images using the Filament Editor function of Amira 5.3 software.

Neuronal tracing was performed in 3D both with the Simple Neuronal Tracing plugin of ImageJ/Fiji^2^ and with The Filament Editor function of Amira 5.3 software.

### Data Analysis

Graphs and data analysis were done with OriginPro 2017 (OriginLab Corporation). Data were analyzed using one-way analysis of variance (ANOVA) with Bonferroni’s test for multiple comparisons. *P* values of ≤0.05 were considered significant. Image stacks were analyzed using both Fiji^2^ and Amira 5.3 software.

## Results

### Custom Made PDMS Cuvettes

Customized PDMS cuvettes for LSFM samples holding were successfully obtained by replicating a negative aluminum (Al) mold realized with tailored geometry by standard machining processes (Figures 1A,B). The simple and efficient method enables the low-cost fabrication of cuvettes of varied dimensions with little difficulty. Furthermore, a single Al mold can create many PDMS replicas, clearly highlighting the “money-saving aspect” of the proposed method. Prior to pouring the PDMS prepolymer to realize the cuvette, the Al templates were functionalized with a PFDTES low-energy coating. The perfluorinated monolayer resulted in fundamental reduction in adhesion between PDMS and the master and to facilitate PDMS peeling off from the central part of the mold, key requirements for an efficient and reliable template. Obtained cuvettes were characterized by surfaces good finishing, which is crucial for avoiding image quality loss due to light scattering phenomena.

In order to minimize possible optical aberration caused by the presence of the cuvette itself along the light-path during imaging, thin sides are desirable. Asking for commercially available quartz cuvettes with thin walls, the thinner wall thickness associated with the required cuvette dimension was of 1 mm. We then chose 1 mm as wall thickness also for our custom PDMS cuvettes. PDMS revealed to be fluid enough to be poured in the 1 mm narrow fissures of the Al mold. Despite the elastomeric properties of PDMS, a wall thickness of 1 mm was enough to give mechanical stability to the overall part and ensure a stable coupling of the cuvette to the dedicated LSFM cuvette holder (Figure 1C). Finally, to verify that no undesired dimensional alterations occurred, swelling tests were performed by immerging overnight PDMS cuvettes in TDE/PBS solutions at different percentages (data not shown). Visually, the created PDMS cuvette appears indistinguishable from the quartz cuvette, even when containing a whole clarified mouse brain (Figures 1C,D).

### Characterization of Light Transmittance Properties and Sample Size After Clearing

**Figure 2:**
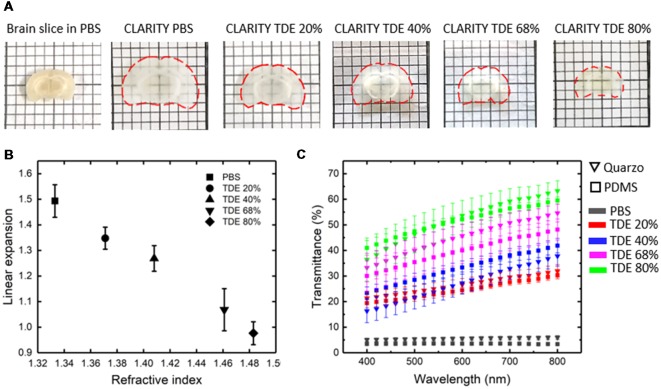
Sample deformation and transmittance. **(A)** Top view images of a coronal brain slice before CLARITY and after CLARITY treatments in different mediums. Slice contour is outlined with dashed red lines. **(B)** Expansion of 1 mm thick brain slices after tissue transformation with CLARITY, and incubation in different TDE/PBS solutions, with respect to the original size (*n* = 4, SD). **(C)** Light transmittance of 1 mm thick brain slices placed inside a Quartz or PDMS cuvette, in PBS before clearing and in different TDE/PBS solution after clearing (*n* = 4, SEM).

Imaging of the whole mouse brain with LSFM needs the brain to be treated with clearing methods in order to make it optically transparent (Richardson and Lichtman, 2015; Silvestri et al., 2016). Among the clearing procedures present in literature, the coupling between CLARITY and TDE clearing (Costantini et al., 2015) represent a versatile option enabling fine tuning of the RI of the cleared specimens. As known, the lipid removal carried out with CLARITY caused sample expansion, which is reverted with the successive treatment with the RI matching solution. We then characterized alterations in sample size after treatments with different TDE/PBS ratio (Figures 2A,B). As expected, after the expansion resulting from CLARITY treatment, a gradual shrinkage was observed upon sample incubation in TDE/PBS as TDE concentration increase. However, the shrinkage is not so prominent to revert the swelling occurring after CLARITY, so that the sample remains expanded with respect to its original size. The concentration of 40% and 68% of TDE in PBS where chosen for characterization as RI matching solution for PDMS (RI 1.41) and quartz (RI 1.46) respectively. At these concentrations we found a linear expansion of 1.27 ± 0.05 and 1.07 ± 0.08 (mean ± SD) for 40% and 68% TDE in PBS, respectively (Figure 2B).

At the same time, the optical properties of samples placed in PDMS and quartz cuvettes, after incubation at different TDE/PBS ratio were measured (Figure 2C). The transmittance curves revealed no significant differences of light transmittance in the visible wavelength range using PDMS or quartz cuvettes, for all the TDE/PBS ratio used as incubation media. Despite the different RI of quartz and PDMS, for both materials, a greater transmittance was revealed with 68% of TDE in PBS. At higher TDE/PBS ratio (80%), a decreased light transmittance was found. We infer that this effect can maybe a consequence of at least two factors: the sample shrinkage observed, and a reduced RI matching between the medium and the hydrogel mesh constituting the samples. In addition, it is not to exclude a browning effect, which is notable by eye after long sample incubation at high TDE percentages. We, however, did not inspect further on that, being concentration values higher than 68% out the range we used for imaging. Although TDE 68% in PBS yields the higher transmittance, a good light penetration was found also for TDE 40%, high enough to perform whole mouse brain acquisition with LSFM (see below).

### LSFM Imaging of Clarified Mouse Brain Using PDMS and Quartz Cuvettes

**Figure 3:**
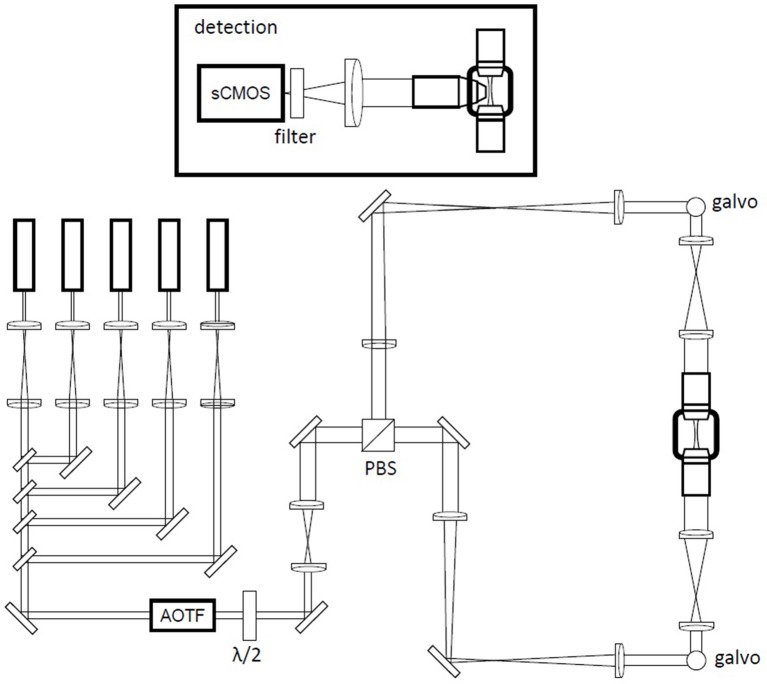
Schematic of the custom-made light-sheet microscope. A battery of five laser lines is multiplexed into a common beam path with long-pass filters. An AOTF (acousto-optical tunable filter) is used as a shutter and for power regulation. Double-sided illumination is generated by rapid scanning of the excitation beam with galvanometric mirrors in two equivalent excitation arms selected by input polarization *via* a half wave plate (λ/2) and polarization beam splitter (PBS) combination. The galvanometric mirrors are mounted above periscopes. Inset: detection path in a wide-field configuration using a specialized objective for direct immersion in high-refractive-index (RI) media and a sCMOS (scientific complementary metal oxide semiconductor) camera.

**Figure 4:**
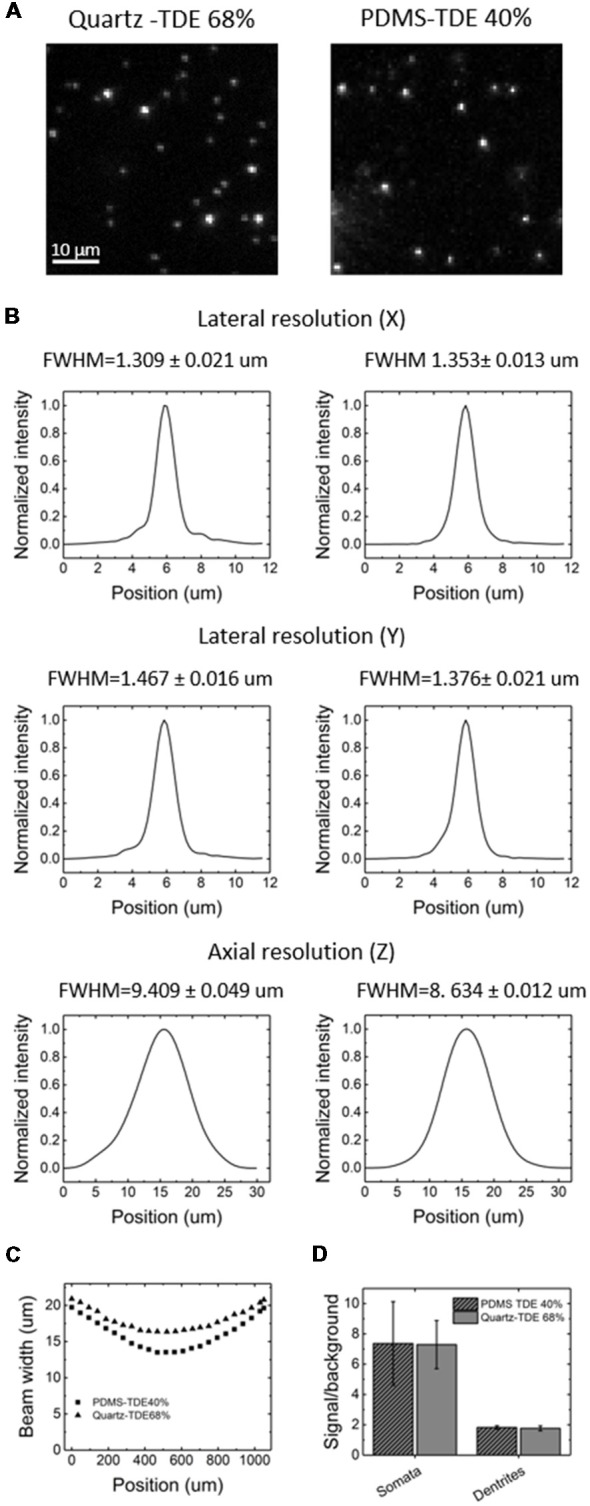
Image quality comparison between PDMS and quartz cuvettes. **(A)** Light-sheet fluorescence microscopy (LSFM) images of fluorescent beads embedded in 2% agar, acquired in quartz (left) or PDMS (right) cuvette in the respective RI matching medium. **(B)** Lateral and axial resolution using PDMS (left) or quartz (right) cuvette, extrapolated from imaging of the fluorescent beads. FWHM, Full-width-at-half-maximum. **(C)** Beam width profile calculated from fluorescence imaging using PDMS or quartz cuvette in their respective RI matching medium. **(D)** Signal-to-background ratio at the level of neuronal somas or dendrites using PDMS or quartz cuvette (*n* = 10, ± SEM).

**Figure 5:**
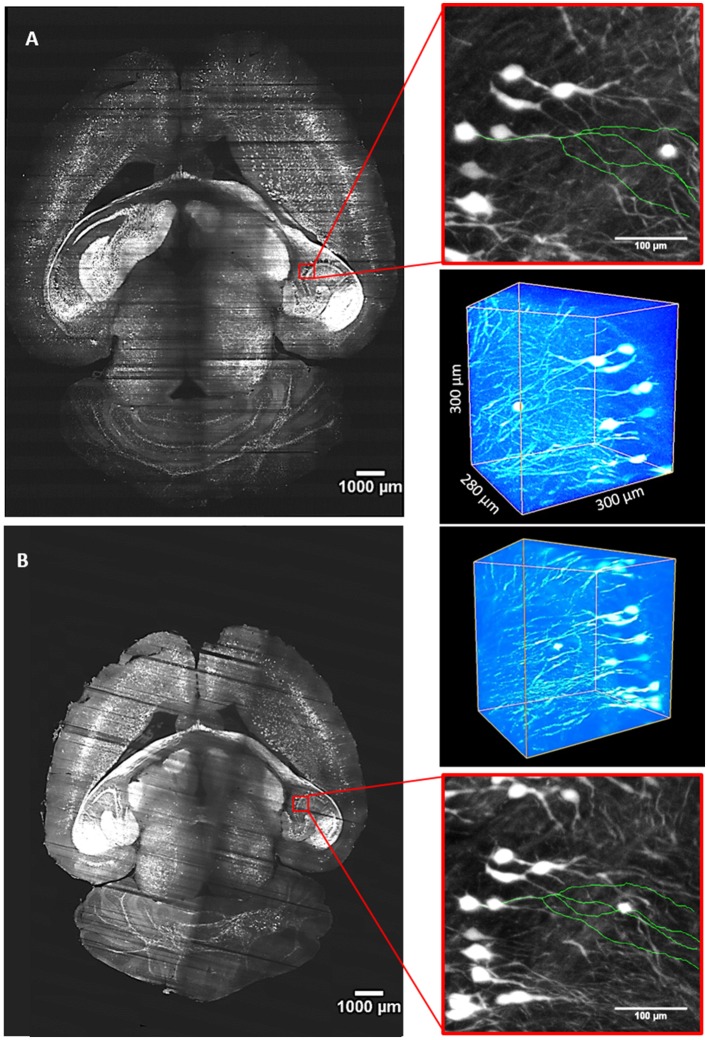
Whole-brain imaging with quartz and PDMS cuvettes. **(A)** Stitched slice from whole *Thy1*-GFP-M mouse brain imaging with LSFM using a custom made PDMS cuvette for sample mounting and TDE 40% in PBS as RI matching solution. **(B)** Imaging of the same brain placed in a quartz cuvette and incubated in TDE 68% in PBS. Red insets are MIPs showing an enlarged view in the hippocampal area. In green, 3D tracings of the same neuron from the two imaging modalities are overlaid on the MIPs. Worth of note is the different brain dimension caused by the different TDE/PBS ratio used for the two imaging sessions. Three-dimensional renderings of ROIs are shown at the upper right and bottom right sides for **(A,B)** panels, respectively.

**Figure 6:**
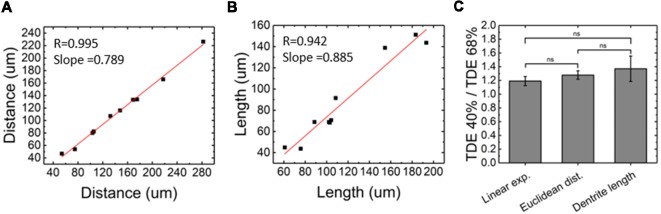
Evaluation of morphological alteration from LSFM images. **(A)** Euclidean distance between neurons measured retrieving the same neurons from LSFM images acquired with Quartz and PDMS cuvettes (*n* = 10). **(B)** Comparison between neuronal processes length measured from LSFM images acquired with Quartz and PDMS cuvettes (*n* = 10). **(C)** Comparison between linear expansion (*n* = 4), euclidean distance between neurons (*n* = 10), and dendrite length (*n* = 10) resulting from TDE 40%/TDE 68% ratios (mean ± SD). ns: not significant.

For our experiments, we used a custom-made setup specifically designed for whole mouse brains imaging, and described in [Müllenbroich et al. (2015); Figure 3]. Before proceeding with imaging of biological samples, we used the setup to ascertain that the use of PDMS cuvette with 40% TDE does not introduce additional aberration affecting resolution (Figures 4A,B). As shown by the curves and the FWHM values obtained from the point spread functions, calculated using fluorescent beads, the resolution does not get worse using the PDMS cuvette. As a further parameter, we compared the laser beam width (Figure 4C). The beam waist (W_0_) of the laser passing through the PDMS cuvette appear slightly thinner (W_0_PDMS = 13.40 μm, W_0_ quartz = 16.34 μm) maybe because of the smaller RI mismatching between the air objective used for excitation and TDE 40%, with respect to TDE 68%. At the same way, the Rayleigh range (Z_R_) appear shorter for imaging with PDMS cuvette (Z_R_PDMS = 452 μm, Z_R_quartz = 582 μm). As last step, a direct comparison of whole mouse brain acquisition with LSFM was performed using quartz and PDMS made cuvettes. Specifically, we use a clarified whole GFP-M mouse brain, a widely used transgenic mouse model in which sparse pyramidal neurons are genetically labeled with a fluorescent marker (Feng et al., 2000). The evaluation of signal-to-background ratio at both somata and dendritic processes result in comparable using PDMS-TDE 40% and Quartz-TDE 68% (Figure 4D). As a practical demonstration of the good image quality achieved, we performed semiautomatic tracing of the same neuron retrieved from the two whole brain acquisitions of the same sample (Figures 5A,B, red insets). As notable, the sample imaged with PDMS cuvette using a TDE/PBS ratio of 40% is larger with respect to the acquisition at 68% TDE/PBS in a quartz cuvette. We believe that the greater sample expansion could represent an advantage in term of resolution of fine structures, as previously demonstrated by the so-called expansion microscopy (Chen et al., 2015). In this technique, the expansion of the hydrogel mesh in which the sample has been transformed after clearing removal is exploited to resolve fine neuronal processes, otherwise undetectable with the same optical methods. For obtaining meaningful information on neuronal distribution and axonal connection, it is important that this size change does not introduce anisotropic deformations. To evaluate if there are relevant morphological alterations between the use of the two images modality, affecting investigations of neuronal cytoarchitecture and/or neuronal projection pathways, 3D measurements of Euclidean distance between neurons, and tracing of neuronal processes were performed (Figures 6A,B). The high correlation coefficients (*R* = 0.995, *R* = 0.942 for Euclidean distance and dendrite branches length, respectively) suggest that the shrinkage occurring during TDE incubation is isotropic. In both cases, the slope of the fitted curves bends towards the PDMS axes, in agreement with the different sample dimensions, although with a slight difference between Euclidean distance between neurons (slope = 0.789 ± 0.019) and dendrite length (slope = 0.885 ± 0.077). In addition, we verified if the values found with the different analysis were consistent. For the latter purpose, the ratios resulting from measures of Euclidean distance of the same neurons (retrieved from PDMS and quartz cuvettes acquisitions), as well as the ratios found analyzing dendrite lengths, were compared both between them and with the linear expansion values obtained from top view photos of the samples (Figure 6C). The non-significant differences emerging from statistical analysis confirm a consistency between the measurements carried out. This finding supports the hypothesis that an intact cytoarchitectonic structure is maintained during the described sample treatments, and that optical aberrations affecting such investigations do not occur with the custom PDMS cuvettes created.

## Discussion

The application of light-sheet microscopy for imaging of biological samples entailed dedicated strategies for sample mounting to be devised. Embedding in agarose, samples fastening with glues, tips, or hooks have been used, which however result in compromised image quality or sample damage. Tailor-made quartz cuvettes allow stable sample placement, without physical damage and without optical aberrations upon sample treatment with RI matching procedures. The price for making custom quartz cuvettes is however high (roughly 600 Euros per cuvette, depending on size).

We proposed a cheap alternative consisting of the use of custom PDMS cuvettes. PDMS (Sylgard 184 silicone elastomer) demonstrated to be suitable for the creation of custom cuvettes with thin walls (1 mm thickness), thanks to its relative low viscosity before curing and hardness after the curing reaction. The light transmittance properties appeared suitable for imaging employing the visible light spectra and comparable with the transmittance of samples placed in a quartz cuvette. Whole mouse brain imaging with LSFM also yielded similar image quality, demonstrating its applicability in the acquisition of morphological information from biological specimens. The brain was treated with a solution of 40% TDE/PBS for RI matching, which differs from quartz RI. A consequence of the different percentage of TDE used is that the brain remains enlarged with respect to its original dimension. We speculate that this feature could be advantageous for a clearer distinction of fine morphological structures. Worth of note, the change in sample size is isotropic, so no morphological alterations are introduced. The increase in dimensions, however, is modest and does not have a tremendous impact on data volume generated, which must be kept in consideration when acquiring whole brain volumes.

The use of PDMS could also be helpful in association with other microscopy techniques. Since PDMS shows high transmittance in the near infrared (Zahid et al., 2017), it is in principle compatible with two-photon excitation. Two-photon light-sheet microscopy devices have been designed and showed to be advantageous in term of resolution in case of small scattering samples (Truong et al., 2011; Lavagnino et al., 2013). Also, the availability of sample holders with a RI lower than quartz or glass may allow reducing aberration artifacts in super-resolution light-sheet microscopy (Cella Zanacchi et al., 2011; Chen et al., 2014). Indeed, standard mounting in agarose gel might introduce some astigmatism (Cella Zanacchi et al., 2011), whilst inverted LSFM systems—which do not require sample mounting in a holder—are not always available to end users.

Furthermore, the application of such material for customized sample holder can be extended to other optical sectioning microscopy modalities, i.e., point-scanning confocal or two-photon microscopy. Although the slow image acquisition rate of these methodologies makes them not suitable for imaging of large volumes, small sample portions can be acquired at higher resolution with respect to light-sheet microscopy. Tailor-made sample holders would then be useful in experimental designs exploiting correlative approaches in which classical tissue section positioning on glass slides is not applicable because of the need to maintain sample integrity for successive analysis. Beyond the mentioned possibilities, a further application, simply consisting in cover glass replacement with a thin PDMS film, could be adopted in association with 40%TDE as the clearing agent and RI matching solution.

We do not neglect that the generation of a mold can pose some limitations. For instance, the lateral side walls of the molds should be characterized by smooth surfaces otherwise affecting the image. Milling capabilities should then be appropriate to generate a plan and smooth mold surfaces. In the light of the above considerations, we suppose that 3D printing processes could be exploited for the rapid fabrication of molds as they show crucial advantages in comparison to traditional subtractive manufacturing approaches, such as freedom of design and tool-less fabrication (Gibson et al., 2010). As a matter of fact, 3D printing represents a consolidated technology enabling for the fabrication of rapidly configurable and highly customizable parts (polymeric or metallic) with achievable resolution down to the submicron scale (Antonov et al., 2004; Chang et al., 2018).

Finally, as microscopy technologies for biological investigations rapidly evolve, a strategy to create customizable sample holders will be beneficial for future advancement requiring new sample mounting approaches.

## Author Contributions

AD and LS designed the study. CC optimized the replica molding process. AF cleared the sample used for light-sheet imaging. AD and CC carried out light-transmittance measurements. AD and AF performed image acquisitions with the light-sheet microscope. MM wrote the MatLab code used for laser beam profiles analysis and made the schematic of the light-sheet microscope. FP supervised the project. AD analyzed the data, prepared the figures and wrote the manuscript, with inputs from all the other authors.

## Conflict of Interest Statement

The authors declare that the research was conducted in the absence of any commercial or financial relationships that could be construed as a potential conflict of interest.

## Footnotes

^1^http://fiji.sc/Fiji

^2^http://fiji.sc/Fiji

## Supplementary Material

DATA SHEET 1 CAD models for the aluminum mold realized by standard machining processes. The exploded view shows that the mold consists of four side parts and a central one assembled on a single platform.
