# Supplementary figures and images for: Tailored Sample Mounting for Light-Sheet Fluorescence Microscopy of Clarified Specimens by Polydimethylsiloxane Casting
Source: Front Neuroanat. 2019 Mar 27;13:35. doi: 10.3389/fnana.2019.00035 (PMC6445857; doi:10.3389/fnana.2019.00035)

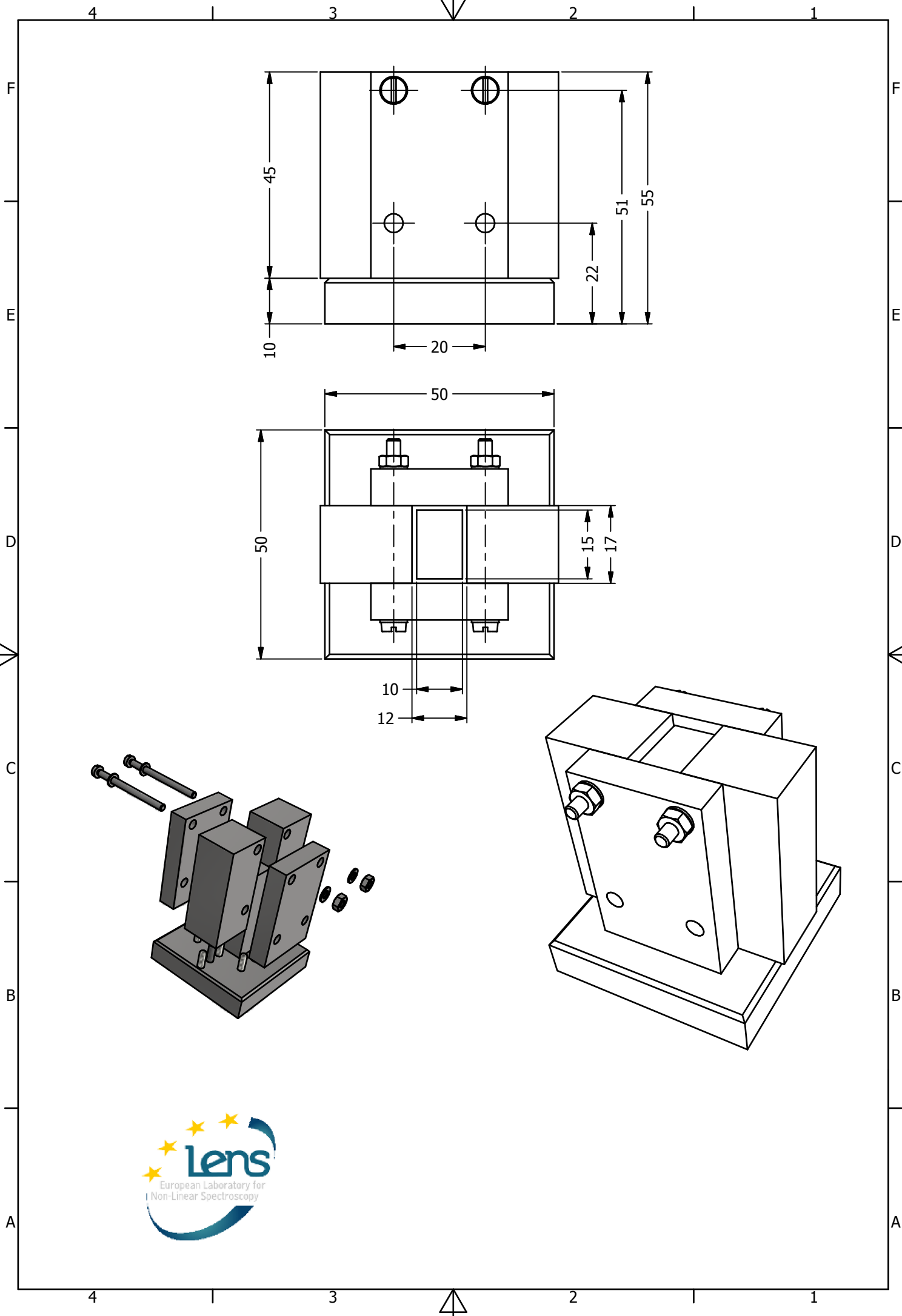

Supplement: DATA SHEET 1 — CAD models for the aluminum mold realized by standard machining processes. The exploded view shows that the mold consists of four side parts and a central one assembled on a single platform. [file Data_Sheet_1.ZIP › CAD_model/2DCAD_model.pdf]
